# Development of a *Trypanosoma cruzi* strain typing assay using MS2 peptide spectral libraries (Tc-STAMS2)

**DOI:** 10.1371/journal.pntd.0006351

**Published:** 2018-04-02

**Authors:** Gilberto Santos de Oliveira, Rebeca Kawahara, Livia Rosa-Fernandes, Simon Ngao Mule, Carla Cristi Avila, Marta M. G. Teixeira, Martin R. Larsen, Giuseppe Palmisano

**Affiliations:** 1 Department of Parasitology, Institute of Biomedical Sciences, University of São Paulo, São Paulo, Brazil; 2 Department of Biochemistry and Molecular Biology, University of Southern Denmark, Odense, Denmark; Albert Einstein College of Medicine, UNITED STATES

## Abstract

**Background:**

Chagas disease also known as American trypanosomiasis is caused by the protozoan *Trypanosoma cruzi*. Over the last 30 years, Chagas disease has expanded from a neglected parasitic infection of the rural population to an urbanized chronic disease, becoming a potentially emergent global health problem. *T*. *cruzi* strains were assigned to seven genetic groups (TcI-TcVI and TcBat), named discrete typing units (DTUs), which represent a set of isolates that differ in virulence, pathogenicity and immunological features. Indeed, diverse clinical manifestations (from asymptomatic to highly severe disease) have been attempted to be related to *T*.*cruzi* genetic variability. Due to that, several DTU typing methods have been introduced. Each method has its own advantages and drawbacks such as high complexity and analysis time and all of them are based on genetic signatures. Recently, a novel method discriminated bacterial strains using a peptide identification-free, genome sequence-independent shotgun proteomics workflow. Here, we aimed to develop a *Trypanosoma cruzi* Strain Typing Assay using MS/MS peptide spectral libraries, named Tc-STAMS2.

**Methods/Principal findings:**

The Tc-STAMS2 method uses shotgun proteomics combined with spectral library search to assign and discriminate *T*. *cruzi* strains independently on the genome knowledge. The method is based on the construction of a library of MS/MS peptide spectra built using genotyped *T*. *cruzi* reference strains. For identification, the MS/MS peptide spectra of unknown *T*. *cruzi* cells are identified using the spectral matching algorithm SpectraST. The Tc-STAMS2 method allowed correct identification of all DTUs with high confidence. The method was robust towards different sample preparations, length of chromatographic gradients and fragmentation techniques. Moreover, a pilot inter-laboratory study showed the applicability to different MS platforms.

**Conclusions and significance:**

This is the first study that develops a MS-based platform for *T*. *cruzi* strain typing. Indeed, the Tc-STAMS2 method allows *T*. *cruzi* strain typing using MS/MS spectra as discriminatory features and allows the differentiation of TcI-TcVI DTUs. Similar to genomic-based strategies, the Tc-STAMS2 method allows identification of strains within DTUs. Its robustness towards different experimental and biological variables makes it a valuable complementary strategy to the current *T*. *cruzi* genotyping assays. Moreover, this method can be used to identify DTU-specific features correlated with the strain phenotype.

## Author summary

Chagas disease is one of the most important neglected diseases with an estimated number of 6–7 million infected individuals, the majority living in Central and South America. The *Trypanosoma cruzi* (*T*.*cruzi*) protozoan parasite is the etiological agent of Chagas disease. *T*.*cruzi* is highly genetically diverse and a new nomenclature assigned each strain to seven genetic groups (TcI-TcVI and Tcbat), named Discrete Typing Units (DTUs), based on their biochemical, immunological and phenotypical characteristics. *T*.*cruzi* DTUs have been correlated to diverse clinical outcomes highlighting the importance of molecular epidemiological screens. Despite the development of *T*.*cruzi* typing methods based on genetic signatures, each method presents its own advantages and challenges. The work presented here shows the application of mass spectrometry for *Trypanosoma cruzi* Strain Typing Assay using MS^2^ peptide spectral libraries (Tc-STAMS2). The novelty of the method is based on the use of peptide fragmentation spectra as strain-specific fingerprints to classify and identify DTUs. Initially, a spectra library is generated from characterized *T*.*cruzi* strains. The library is subsequently inspected using MS/MS spectra from unknown strains and confidently assigned to a specific strain in an automated and computationally-driven approach. The Tc-STAMS2 method was challenged to test several variables such as sample type and preparation, instrument setup and identification platform. Tc-STAMS2 provided high confidence and robustness in *T*.*cruzi* strain typing. The Tc-STAMS2 method represents a proof-of-concept of a complementary strategy to the current DNA-based *T*. *cruzi* genotyping methods. Moreover, the method allows the identification of strain-specific features that could be related to the biology of *T*.*cruzi* strains and their clinical outcomes.

## Introduction

Chagas disease also known as American trypanosomiasis affects around 6–7 million people especially in Latin America [1]. The etiologic agent of Chagas diseases is the protozoan *Trypanosoma cruzi* [2] that infects several mammalian hosts and is primarily transmitted through the contamination with feces of triatomine bugs. Besides, congenital, blood transfusions, transplants and ingestion of contaminated foods represent other ways of infection [3]. Chagas disease is characterized by an acute and chronic phase. The acute phase lasts a few weeks and may present mild symptoms such as fever and swelling around the site of infection[4]. The chronic phase is in general lifelong and asymptomatic. However, 20–30% of patients develop cardiac or gastrointestinal complications[3].

*T*. *cruzi* is highly genetically diverse. In order to standardize the nomenclature facilitating the communication among scientists, *T*. *cruzi* strains were divided into six (Tc I-VI) *discrete typing units* (DTUs) plus Tcbat, a novel strain associated with bats. Each group represents a set of isolates that are genetically similar and can be identified by common immunological, biochemical, pathological and molecular markers [5]. *T*. *cruzi* strains characterization is extremely important to understand different epidemiological and pathological characteristics such as geographical distribution and clinical outcomes [6]. Several techniques have been introduced to improve the genotyping. In particular, the genetic diversity of *T*. *cruzi* was first recognized by multilocus enzyme electrophoresis (MLEE) and restriction analysis of kinetoplastid DNA minicircles [7, 8]. Currently, the methods used for typing of *T*. *cruzi* strains are based on polymorphism of the mini-exon gene (Spliced Leader) and the 24Sα and 18S ribosomal RNA [9]. Other assays involve the analysis of complex electrophoretic patterns generated by restriction polymorphisms of PCR amplified genomic DNA [10–12]. These methods are able to discriminate *T*.*cruzi* strains, but they are work and time consuming and the interpretations of the results may be misleading. Multiplex Real-Time PCR Assay Using TaqMan Probes proved useful to determine DTUs of cultured *T*.*cruzi*, vector and blood samples from patients in acute infection [10, 12, 13].

Proteomics methods using mass spectrometry have emerged as a powerful strategy to discriminate bacteria and have been established as a valuable alternative to DNA-based bacterial identification. Indeed, MALDI-TOF MS generates an intact protein profile that is compared to a MS database of known species. This method is easy to perform, rapid and provides accurate results and has been introduced as routine in most hospitals [14]. However, this method is currently not able to distinguish at the strain level. Shotgun sequencing of peptides, derived from enzymatically digested proteins, using LC-MS/MS in combination with database search of *in silico* digested proteins derived from publicly available protein sequence has been proposed as a method to discriminate bacterial strains such as *Helicobacter pylori* and *Yersinia persis [15].* This approach has great discrimination power compared to MALDI-TOF MS since it allows the identification of thousands of peptides with higher dynamic range. However, this method suffers from lack of suitable databases since many species have not been sequenced. Recently, a novel method based on MS/MS spectral matching has been shown to identify the blood meal of ticks (*Ixodes scapularis*) [16] and has also been applied to discriminate *E*.*coli* strains from different isolates [15].

Few studies have explored the possibility of using mass spectrometry as a diagnostic tool for Chagas disease. Using mass spectrometry, Ndao M. et al. [17] were able to identify full-length Apo1 as a serum biomarker of chronic Chagas disease patients using a SELDI-TOF approach. Despite several proteomic studies to understand the molecular features of *T*. *cruzi* in different biological conditions [18–22], to date, there is no report on the use of mass spectrometry for assaying various *T*. *cruzi* strains.

A pioneer work developed a peptide identification-free shotgun proteomics workflow to trace the vertebrate host that a tick (*Ixodes scapularis*) was feeding on [16]. This workflow used MS/MS spectral matching. The same strategy, named UNID, was used to profile bacterial strains [15]. In this study, we developed a platform to discriminate *T*. *cruzi* strains using MS/MS peptide spectral matching (Tc-STAMS2). The method was able to discriminate *T*. *cruzi* strains from different origins with high sensitivity and accuracy. The method was robust towards sample preparation and instrumental parameters, such as peptide purification, chromatographic gradient time, peptide fragmentation techniques and MS instruments. The MS/MS spectra were subjected to database search. More than 4000 proteins were identified in the combined six DTUs strains analyzed.

A total of 1096 proteins were differentially expressed between the six DTUs and multivariate analysis allowed the discrimination of *T*. *cruzi* strains using the quantitative MS signal. In conclusion, this study describes a mass spectrometry-based method to discriminate *T*. *cruzi* strains. The Tc-STAMS2 method represents a proof-of-concept of an alternative strategy to DNA-based *T*. *cruzi* genotyping. Further studies are needed to show its applicability to biofluids in clinical isolates.

## Methods

### *T*. *cruzi* cultures

Epimastigote forms of *T*. *cruzi* cultivated in LIT (Liver Infusion Tryptose) supplemented with 10% fetal bovine serum [23] at 28°C of exponential culture phase were employed in the present study. DTU classification of all *T*.*cruzi* strains were confirmed by sequencing [24]. Only validated DTUs strains were used in this study (**Table 1**).

**Table 1:** Cultured stocks representing the known *T*. *cruzi* lineages (DTUs) used to build the MS/MS library and to validate the method.

Trypanosomatid	DTUs	Strains	Form	Culture médium
*T*. *cruzi*	DTU-I	Sylvio X10 cl1	Epimastigote	LIT—Liver Infusion-Tryptose supplemented with 10% fetal bovine serum at pH 7,2; 28 °C [23].
	DTU-I	Sylvio X10/4*		
	DTU-I	G*		
	DTU-II	Y		
	DTU-II	Esmeraldo*		
	DTU-III	M6241 cl6		
	DTU-III	3869*		
	DTU-IV	CanIII cl1		
	DTU-IV	José Júlio clone*		
	DTU-V	MN cl2		
	DTU-V	NR cl3*		
	DTU-VI	CL Brener		
	DTU-VI	CL14*		

*Strains added to validate the Tc-STAMS2 methodology for intra-DTU strain discrimination.

### Cell growth challenging test

*T*. *cruzi* cells from the Sylvio X10 cl1 (DTU-I) strain were collected in the exponential and stationary phase and processed as described below. Two biological replicates for the stationary (St_1 and St_2) and exponential (Exp_1 and Exp_2) growth phase were analyzed.

### Sample preparation and nLC-MS/MS analysis

*Protein extraction and digestion*. Epimastigote cells (5x10^8^) were washed three times in phosphate buffered saline (PBS), pH 7.2 (8,000g for 10 minutes at room temperature), re-suspended in 400 μL of lysis buffer (7M urea, 2M thiourea, 1 mM DTT and protease inhibitors (Amersham) and incubated under stirring for 30 minutes to solubilize the proteins. Proteins were reduced with 10 mM DTT (DL-Dithiothreitol–Sigma-Aldrich), alkylated with 40 mM iodoacetamide (Sigma-Aldrich), digested with trypsin (Promega) in the ratio 1:50 (μg trypsin/μg protein) in 50 mM ammonium bicarbonate solution at 37°C overnight. The reaction was stopped with 1% formic acid (less than pH 3) and then the sample was desalted with C18 columns (StageTips). Four biological replicates were prepared for each DTU. Blind test samples A (DTU-III) and B (DTU-I) were prepared using a minimum of three biological replicates according to the protocol described above.

### Sample preparation for the challenging tests

Peptide desalting was performed in acid (acid 1 and acid 2) and basic (basic 1 and basic 2) conditions. In particular, for the acid purification tryptic peptides were acidified with 0.1% TFA (pH 3) and loaded onto an acid-activated StageTip microcolumn before being eluted with 50% acetonitrile: 0.1% TFA. For the basic purification, tryptic peptides were dissolved in 0.1% ammonia water (pH 10) and loaded onto a base-activated StageTip before being eluted with 50% acetonitrile: 0.1% ammonia water. The eluted peptides were lyophilized and analyzed by liquid chromatography tandem mass spectrometry (LC-MS/MS).

### Nano LC-MS/MS analysis

Peptides were separated by Reprosil-Pur C18-AQ column (3μm; Dr. Maisch GmbH, Germany) using Easy nano-LC HPLC (Proxeon, Odense, Denmark). The HPLC gradient was 0–34% B solvent (A = 0.1% formic acid; B = 90% ACN, 0.1% formic acid) in 70 min at a flow of 250 nL/min. The MS analysis was performed using the LTQ-Orbitrap Velos (Thermo Scientific, Bremen, Germany). The mass range was 400–1500 m/z at a resolution of 30,000 at 400 m/z for a target value of 1e^6^ ions. For each MS scan, collision induced dissociation (CID) fragmentation was performed on the 20 most intense ions in the linear iontrap. The parameters for data acquisition were: activation time = 15 ms, normalized energy = 35, Q-activation = 0:25, exclusion = available with repeat count 1, exclusion duration = 30s and intensity threshold = 30.000, target ions = 2e^4^ [25]. All raw data have been submitted to PRIDE archive (https://www.ebi.ac.uk/pride/archive/), project accession: PXD008088.

### Sample amounts, chromatographic gradients and MS/MS fragmentation types used for the challenging test

The robustness of the Tc-STAMS2 method was tested using different parameters: 1) sample amounts, 2) chromatographic gradients and 3) MS fragmentation techniques. For the different sample amounts, 0.5μg and 1μg, were loaded onto the analytical column before MS analysis and named low and high, respectively. The chromatographic elution time was set to 20, 70 and 130 min from 0–34% B solvent at 250nL/min. CID fragmentation was used to develop the Tc-STAMS2 method. Higher energy collision induced dissociation (HCD) was evaluated as alternative peptide fragmentation type on tryptic peptides separated on a 70min chromatographic gradient. For the HCD fragmentation, each MS scan was acquired at resolution of 30,000 FWHM followed by 7 MS/MS scan of the most intense ions with an activation time of 0.1 ms and normalized collision energy of 35. The spectral library for each DTU was developed on a LTQ-Orbitrap Velos Pro instrument (Thermo Fisher Scientific) located in the PR group, Department of Biochemistry and Molecular biology, University of Southern Denmark. All the other tests for testing and validating the method were performed on a LTQ-Orbitrap Velos Normal at the Biomass mass spectrometry facility (São Paulo, Brazil).

### Bioinformatics and statistical analyses

*MS/MS spectral library generation and spectral matching*. MS/MS spectral library generation and spectral matching were performed using the SpectraST software (version 4.8) as previously described [15, 16, 26]. In particular, the LC-MS/MS acquired spectra were converted to an open format (mzXML) by MSconvert [27], forming part of the software suite offered by TPP (Trans-Proteomic Pipeline) [28]. SpectraST (version 4.8) was used to build the reference spectral library and perform MS/MS spectral matching [26]. The reference spectral library was generated with three raw files for each one of the six DTUs and one raw file for each DTU was compared against constructed reference library. The first step for the reference spectral library generation involves the application of a threshold at which MS/MS spectra originated from the same peptide precursor ion are combined to create the consensus spectrum. Moreover, low quality spectra are excluded from the library [26]. To determine the spectrum of similarity between the query spectrum and the reference library, SpectraST uses the Spectral Dataset Similarity (SDSS) function [16, 29]. In particular, the unique dot product SDSS, abbreviated as “score” along the text, was chosen as *T*.*cruzi* DTU strain discrimination function and reported [16]. The statistical confidence in the identification of the correct DTU is made by data bootstrap [16]. All the spectral matching experiments reported below had a bootstrap of 1 unless reported.

DiagnoProt software was used also to match the MS/MS spectra of unknown samples against a database of genotyped *T*. *cruzi* strains [21]. Default parameters for creating the spectral database were used: similarity threshold 0.70, precursor tolerance 4.50; activation type CID; minimum number peaks: 50; minimum relativity intensity: 0.01; minimum retention time 10.00; bin offset 0.40; bin size: 1.0005; minimum bin m/z 200.00; maximum bin m/z 1700.00. The spectral database was used to match the identity of each unknown sample.

### Database searches

The raw LC-MS/MS files were analyzed using: Proteome Discoverer, MaxQuant and the Trans-Proteomic Pipeline. Proteome Discoverer v2.1 (Thermo Scientific) was used with the *T*. *cruzi* database using Mascot and Sequest. The searches in the database were conducted with the following parameters: precursor mass tolerance of 20 ppm; MS/MS mass tolerance 0.5 Da (CID data). Trypsin was selected as enzyme and carbamidomethyl cysteine as fixed modification. The variables modifications were oxidation of methionine and deamidation (NQ). Shared peptide sequences were grouped as grouped accessions proteins. The False Discovery Rates (FDR) was calculated using the algorithm Percolator with *q* equal or less than 0.01. Protein FDR was calculated in the Proteome Discoverer software and kept below 1%.

The raw files were also processed using the MaxQuant [30] version 1.2.7.429 and the MS/MS spectra were searched using the Andromeda search engine [31] against the Uniprot *T*. *cruzi* Protein Database (release July 11, 2017; 51,738 entries). The initial maximal allowed mass tolerance was set to 20 ppm for precursor and then set to 4.5 ppm in the main search and to 0.5 Da for fragment ions. Enzyme specificity was set to trypsin with a maximum of two missed cleavages. Carbamidomethylation of cysteine (57.02 Da) was set as a fixed modification, and oxidation of methionine (15.99 Da), deamidation (NQ) and protein N-terminal acetylation (42.01 Da) were selected as variable modifications. Bioinformatics analysis was performed using the software Perseus v.1.5.2.6 [30] available in the MaxQuant environment and reverse and contaminant entries were excluded from further analysis. Protein FDR was calculated in the MaxQuant software and kept below 1%. Label Free Quantification (LFQ) intensity values were considered to relatively compare the abundance of proteins present in the different DTUs.

Trans-Proteomic Pipeline software suite was also used to search raw files converted to mzXML [13]. The mzXML files were searched by the Comet search algorithm embedded into the TPP platform [14]. Peptide and protein FDR was estimated using the PeptideProphet and ProteinProphet algorithm embedded in the Trans-Proteomic Pipeline [32]. Identifications with less than 1% FDR were kept.

Raw data from human placental tissue [33] and *T*. *vivax* (Meta, BSF1 and EP1) [22] were obtained from the public MS spectra databank PRoteomics IDEntifications (PRIDE) and used for the negative control test.

### Statistical analyses

Label-free quantified peptides/proteins were analysed by the Perseus software [18]. Significantly regulated features with a p value less than 0.05 corrected with the Bonferroni post-hoc test were used to cluster the different DTUs. Hierarchical clustering of significantly regulated proteins/peptides was performed using the Z-score calculation on the log2 intensity values and it was represented as a heat map. The Principal Component Analysis (PCA) was performed using the same procedure described above in Perseus software. In addition, for generation of Venn diagram we used Venn Diagrams (http://bioinformatics.psb.ugent.be/webtools/Venn/ Bioinformatics & Evolutionary Genomics).

## Results

### Tc-STAMS2 strategy allows DTUs discrimination

**Fig 1:**
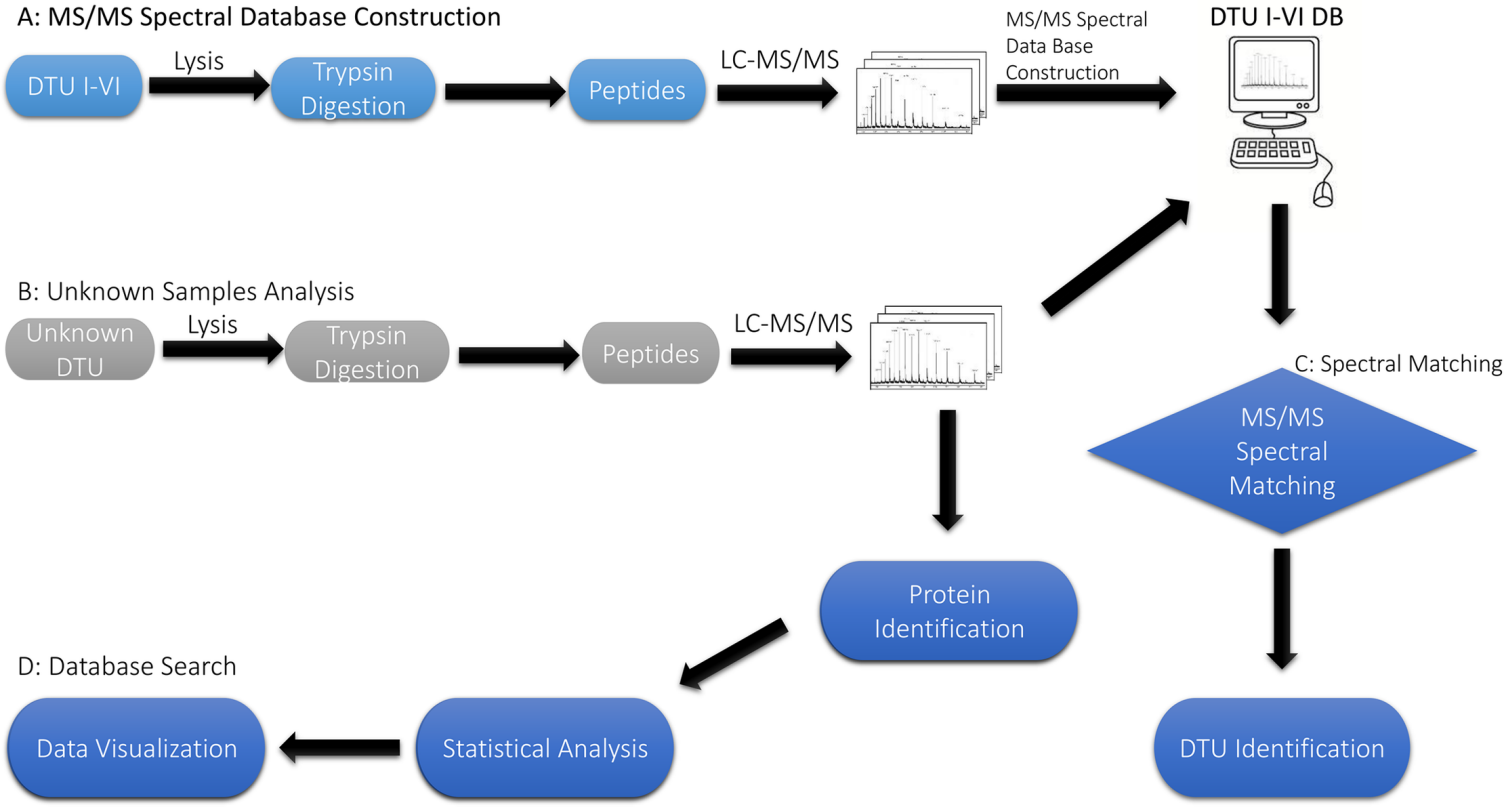
Tc-STAMS2 workflow. (A) Proteins were extracted from epimastigote *T*. *cruzi* cells, digested using trypsin and subjected to nLC-MS/MS analysis. MS/MS spectra were clustered and merged to generate a reference mass spectral library using SpectraST (http://tools.proteomecenter.org/wiki/index.php?title=Software:SpectraST) or DiagnoProt (http://patternlabforproteomics.org/diagnoprot) software. (B) The same steps described in panel A were used for the unknown samples. In particular, the unknown *T*.*cruzi* strains were lysed, proteins were extracted and digested using trypsin. Tryptic peptides were analyzed by nLC-MS/MS. C) The MS/MS spectra from unknown *T*.*cruzi* strain samples were compared with the reference MS/MS spectral library in order to assign an unknown sample to a particular *T*.*cruzi* strain. The A, B and C steps have been used through the text in all spectral matching analyses. (D) MS/MS data were searched against the Uniprot *T*.*cruzi* protein database to identify and quantify proteins in each strain. Three computational platforms were used: MaxQuant (30, 31), Proteome Discoverer v2.1 (Thermo Fisher) and Trans-Proteomic Pipeline (13, 14).

In this study, the combination of mass spectrometry and computational approaches was used to develop a method for *T*. *cruzi* DTU discrimination, named Tc-STAMS2. A schematic overview of the *T*. *cruzi* DTUs identification using MS/MS spectra from tryptic peptides is summarized in **Fig 1**. A reference spectral library was built using a total of 586513 unique MS/MS spectra of tryptic peptides derived from three raw MS files of each one of the six *T*. *cruzi* strains (**Fig 1A**). Each *T*. *cruzi* strain was processed and acquired in four biological replicates. MS/MS spectra acquired from three replicates of each DTU were used to build the reference spectral library using SpectraST software [26]. Following the construction of the reference MS/MS library, proteins from unknown *T*. *cruzi* strain samples were extracted and digested with trypsin before being analyzed by nLC-MS/MS (**Fig 1B**). A LC-MS profile of four replicates of DTU-I is reported in **S1 Fig** and the Pearson correlation score indicates high similarity between the different runs. The LC-MS chromatographic profile of the tryptic peptides belonging to the six *T*.*cruzi* strains shows high similarity between the different DTUs, **S2 Fig.** MS/MS spectra from different DTUs were subjected to spectral matching comparison with the library using SpectraST software [19]). The identification was made by finding the reference library with the highest similarity to the sample spectral dataset, in this case, the different DTUs, to be tested. Unique dot product SDSS score was used to provide a quantitative similarity measure between two spectral datasets (**Fig 1C**) [16]. MS/MS spectra were searched against the *T*. *cruzi* proteome database as described below (**Fig 1D**).

Based on the similarity scores between the MS/MS spectra from an unknown sample with the mass spectral library, the Tc-STAMS2 was able to differentiate and accurately identify the different DTUs. We observed that the score values were between 0.75 to 0.86 for true matches and close to 0 in unmatched cases, as shown in **Table 2**.

**Table 2:** Genotype discrimination based on spectral similarity searches. Six *T*.*cruzi* strains (Sylvio X10 cl1, Y, M6241 cl6, CanIII cl1, MN cl2 and CL Brener) belonging to six DTUs were selected to test the Tc-STAMS2 method. The unique dot product SDSS score is reported along with the number of MS/MS spectra matches (unique/total). Genotypes identified with the highest score are highlighted in gray.

DTUs	DTU-I	DTU-II	DTU-III	DTU-IV	DTU-V	DTU-VI
DTU-I	0.861 (2858/20232)	0.005 (11/13450)	0.008 (24/13419)	0.008 (26/14135)	0.016 (65/13470)	0.013 (33/14469)
DTU-II	0.010 (23/14212)	0.754 (1169/20004)	0.032 (72/14820)	0.023 (62/15126)	0.007 (22/14198)	0.020 (35/16038)
DTU-III	0.006 (18/13177)	0.020 (45/13838)	0.817 (2086/19587)	0.013 (42/13135)	0.017 (42/13689)	0.015 (35/14200)
DTU-IV	0.011 (38/14307)	0.013 (28/14213)	0.010 (32/13412)	0.849 (2648/20199)	0.004 (16/12889)	0.019 (48/14722)
DTU-V	0.008 (35/12560)	0.009 (20/12405)	0.013 (37/12771)	0.004 (16/11687)	0.863 (3086/19777)	0.011 (28/14215)
DTU-VI	0.016 (51/14230)	0.017 (27/14880)	0.013 (32/14101)	0.017 (51/14047)	0.022 (57/14849)	0.800 (1463/20238)

The LC-MS chromatograms obtained for each replicate and for each DTU (**S1 Fig and S2 Fig**) showed high similarity; however, the developed method was capable of differentiating and identifying each of them. In order to rule out the possibility that different growth phases could influence the assignment of the algorithm, Sylvio X10/1 (DTU-I) epimastigotes in the exponential and stationary phase were collected. Independently of the growth phase, the algorithm was able to assign it to the correct DTU, **Table 3**. This demonstrates that the identification method is not affected by the growth phase of the parasite.

**Table 3:** MS/MS spectral matching comparison using different growth phases of Sylvio X10 cl1 (DTU-I) using SpectraST software. St_1—Stationary phase; St_2—Biological replicate stationary phase; Ex—Exponential phase; Ex_2—Biological replicate exponential phase.

Samples	DTU-I	DTU-II	DTU-III	DTU-IV	DTU-V	DTU-VI	CL14 (DTU-VI)
A (St_1)	0.283	0.032	0.068	0.017	0.075	0.032	0.034
A (St_2)	0.288	0.035	0.067	0.015	0.068	0.029	0.030
B (Ex_1)	0.296	0.036	0.057	0.026	0.104	0.058	0.046
B (Ex_2)	0.301	0.041	0.056	0.027	0.099	0.053	0.051

**Fig 2:**
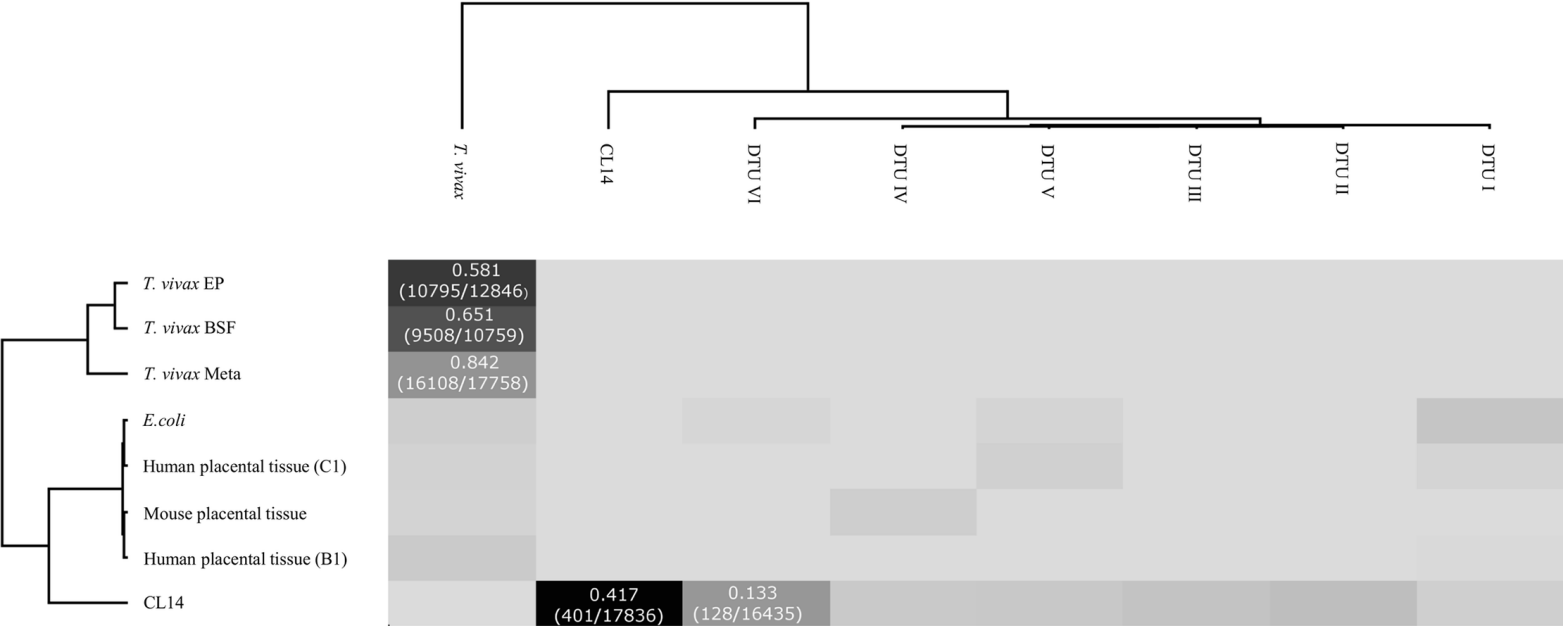
Tc-STAMS2 approach tested against: 1) the CL14 *T*.*cruzi* strain, 2) *T*. *vivax* dataset and 3) LC-MS/MS datasets from *E*.*coli* and human and mouse placental tissues. The sensitivity of Tc-STAMS2 approach was tested for the detection of intra-DTU strains such as CL14 and CLBrener strains belonging to DTU-VI. Moreover, the specificity of Tc-STAMS2 approach was tested for the assignment of MS/MS spectra derived from phylogenetically distant organisms such as mouse and human. In particular, the MS/MS spectral library was built using seven strains belonging to six DTUs such as Sylvio X10 cl1 (DTU-I), Y (DTU-II), M6241 cl6 (DTU-III), CanIII cl1 (DTU-IV), MN cl2 (DTU-V), CL Brener and CL14 (DTU-VI) strains and MS/MS data from *T*. *vivax* (epimastigote, metacyclic and bloodstream forms) were added to the spectral library [22]. Independent LC-MS/MS runs of the different *T*.*cruzi* strains, *T*.*vivax* life stages, human and mouse placenta tissue and *E*.*coli* were compared against the MS/MS spectral library using SpectraST software. Intra-DTU discrimination was achieved for CL14 and CL Brener and no assignment was made for the E.coli, mouse and human samples. MS/MS spectra from *T*.*vivax* were assigned specifically to *T*.*vivax* without identification of *T*.*cruzi*.

Moreover, the performance of the Tc-STAMS2 spectral matching approach were tested to correctly identify MS/MS data sets from: 1) a *T*. *cruzi* strain that is known to belong to DTU-VI (CL14) [5] but was not included in the spectral library, 2) from a species phylogenetically related, such as *Trypanosoma vivax* and 3) from species with completely distant genome (ex: human, *E*. *coli*, mouse). For this analysis, a new MS/MS library was constructed, using the MS/MS spectra from the six DTUs, including *T*. *cruzi* CL14 and *T*. *vivax* in metacyclic stage (meta1 e meta2). Firstly, MS/MS spectra from *T*. *cruzi* strains CL14 were compared with the library and the similarity score matched to the CL14 (score = 0.417). Interestingly, although the similarity scores of CL14 with DTUs I to V were close to zero, the similarity between CL14 and DTU-VI was comparatively high (score = 0.133), indicating that many spectra MS/MS of CL14 are shared with DTU-VI (CL Brener), as shown in **Fig 2**. It should be noted that the LC-MS chromatographic profiles of the CL14 and CL Brener strains have very high similarity, **S3 Fig**. However, the Tc-STAMS2 was able to clearly differentiate between the two strains within the same DTU.

Subsequently, MS/MS spectra from three different life stages of *T*. *vivax*, Meta3 –metacyclic phase, BSF1 –bloodstream phase and EP1 –epimastigote were compared using the mass spectral library. Based on the similarity scores, the Tc-STAMS2 was able to correctly identify these samples to *T*. *vivax*, **Fig 2**.

In order to test the method with negative control, MS/MS spectra from unrelated *T*. *cruzi* species such as human, mouse and *E*. *coli* were compared to the library. For these samples, the similarity scores were close to zero, indicating that the similarity scores found between two MS/MS datasets is library-specific and not random, **Fig 2**.

**Fig 3:**
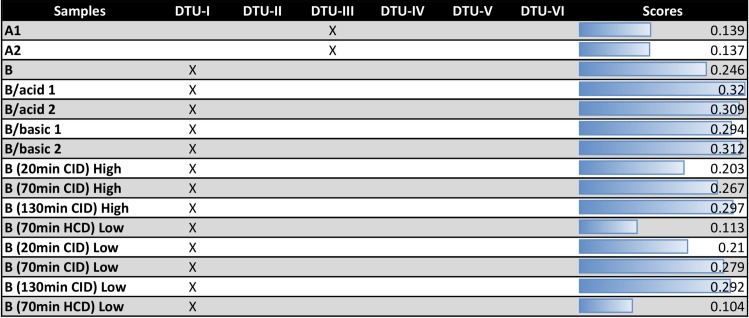
Tc-STAMS2 was tested for its robustness towards technical and experimental variations. Initially, the Tc-STAMS2 approach was tested for inter-laboratory comparison. Two unknown *T*.*cruzi* strains (A and B) were processed as described in the step B of Fig 1 and acquired using the EasynLC coupled to LTQ-Orbitrap Velos mass spectrometer located in the CEFAP mass spectrometry facility at the University of Sao Paulo, Sao Paulo, Brazil. The MS/MS spectral library was built using Sylvio X10 cl1 (DTU-I), Y (DTU-II), M6241 cl6 (DTU-III), CanIII cl1 (DTU-IV), MN cl2 (DTU-V), CL Brener (DTU-VI) and acquired in the PR group, Odense, Denmark using a similar LC-MS/MS setup (EasynLC coupled to LTQ-Orbitrap Velos). A1 and A2 indicate a biological duplicate of *T*.*cruzi* M6241 cl6 (DTU-III). B is the *T*.*cruzi* Sylvio X10 cl1 (DTU-I). Different sample preparation strategies were used to test the robustness of the Tc-STAMS2 approach such as changing the pH for peptide desalting. B/acid refers to peptides derived from sample B were purified using acidic conditions (0.1% TFA). B/basic refers to peptides derived from sample B were purified using basic conditions (0.1% ammonia). Moreover, different analytical parameters were changed in order to test the robustness of the Tc-STAMS2 approach such as the MS/MS fragmentation type, CID—Collision-Induced Dissociation and HCD—Higher-energy collisional dissociation. Different sample amounts were loaded onto the nano LC column. High and Low indicate 1 and 0.5 ug, respectively. The Tc-STAMS2 approach was robust towards different analytical and experimental challenges.

Moreover, we also evaluated the ability of this strategy to provide correct identification from an independent sample (blind test), which was collected and processed at different days or under different conditions. In particular the different datasets were obtained in: 1) inter-laboratory studies, 2) different sample preparation strategies, 3) different LC gradients and 4) different MS fragmentation methods. To assess the robustness of the Tc-STAMS2 platform, another batch of *T*. *cruzi* strains were processed and acquired using similar chromatographic and MS conditions as described in **Fig 1**in an inter-laboratory study perspective. Indeed, the mass spectra library was built with data acquired in the PR group in Odense, Denmark and the blind samples were acquired in the CEFAP mass spectrometry facility in São Paulo, Brazil. Although the instrument type and conditions were similar, a different chromatographic profile was obtained, **S4 Fig**. However, the biological duplicate unknown samples A1 and A2 from DTU-III matched correctly to DTU-III, **Fig 3**. The unknown sample B from the DTU-I also showed higher similarity scores with the MS/MS spectra library from DTU-I. The sample B was evaluated on different parameters: 1) sample preparation conditions such as acid and basic peptide desalting, 2) different chromatographic gradient such as 20 min, 70 min, 130 min, 3) different fragmentation methods, such as CID or HCD with a chromatographic gradient of 70 min and 4) different sample amount injected into the LC column. Even considering all these technical sources of variation, the similarity scores continued to match correctly to DTU-I showing the robustness of the Tc-STAMS2 towards different experimental conditions.

**Fig 4:**
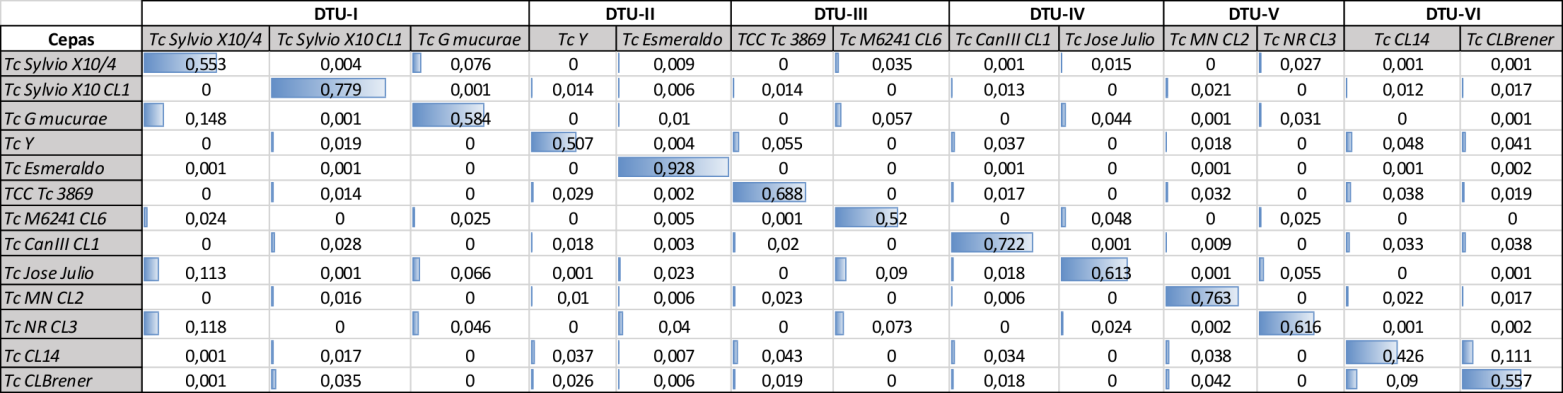
*T*.*cruzi* strain discrimination based on spectral similarity searches. Fourteen *T*.*cruzi* strains (Sylvio X10 cl1, Sylvio X10/4, G, Y, Esmeraldo, M6241 cl6, 3869, CanIII cl1, José Júlio, MN cl2, NR cl3, CL Brener and CL14) belonging to six DTUs were selected to test the Tc-STAMS2 method. The MS/MS spectral library was built using two biological replicates for each strain and one independent replicate was used to search against the library. The unique dot product SDSS score is reported.

**Fig 5:**
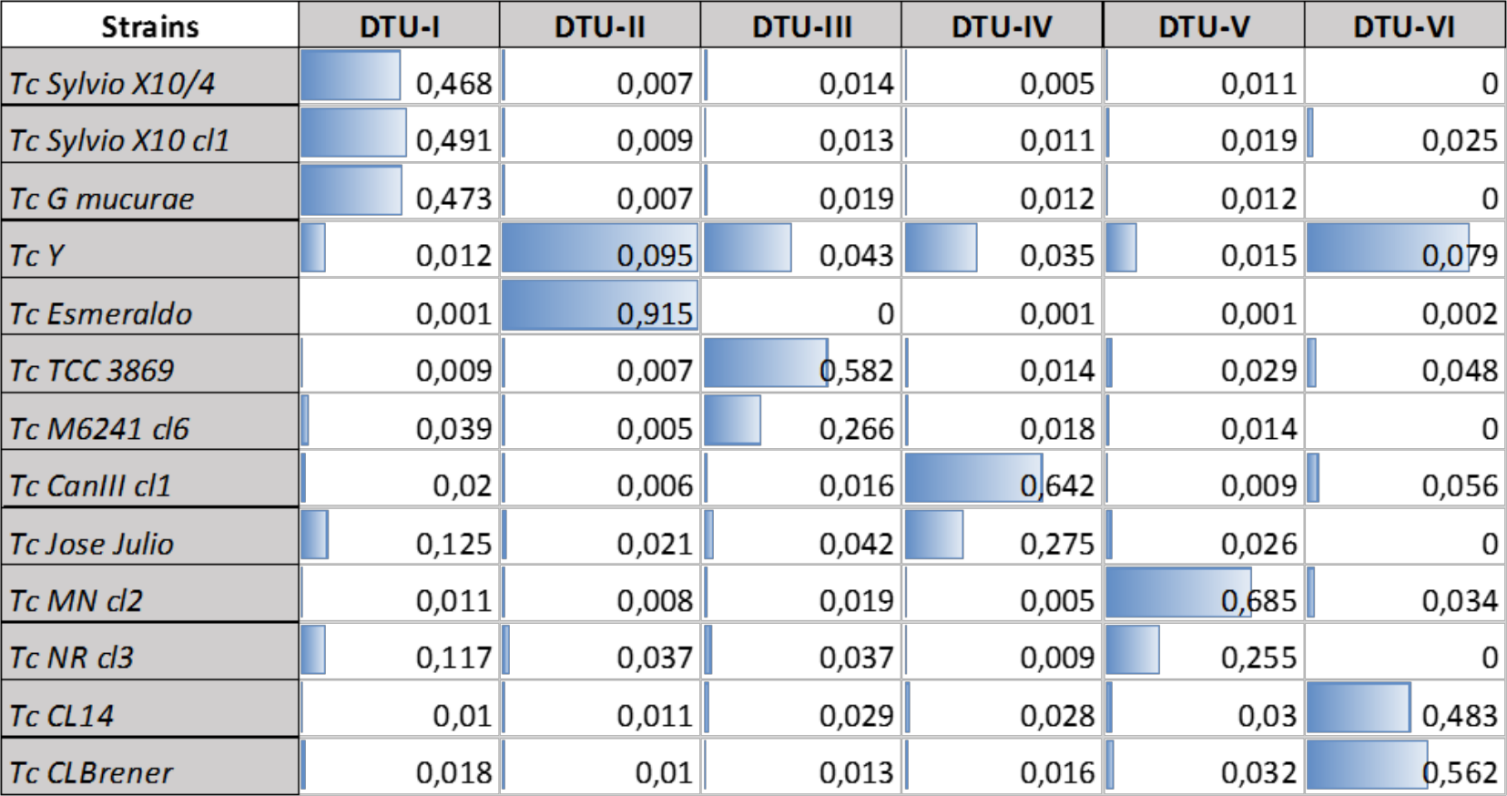
Genotype discrimination based on spectral similarity searches. Fourteen *T*.*cruzi* strains (Sylvio X10 cl1, Sylvio X10/4, G, Y, Esmeraldo, M6241 cl6, 3869, CanIII cl1, José Júlio, MN cl2, NR cl3, CL Brener and CL14) belonging to six DTUs were selected to test the Tc-STAMS2 method. In order to build the MS/MS spectral library, each strain was assigned to the corresponding DTU. Two biological replicates for each strain were used to build the library and one independent replicate was used to match against the library. The unique dot product SDSS score.

In addition, another MS/MS spectral library search software platform, DiagnoProt, was used instead of SpectraST [21]. DiagnoProt was able to differentiate the different DTUs and to associate the CL14 strain with the DTU -VI group as shown for the SpectraST software, **Table 4**. Due to that, two different spectral library search software could be implemented in the Tc-STAMS2 pipeline and used to identify *T*. *cruzi* strains, **Tables 2 and 4**. Furthermore, we tested the Tc-STAMS2 method using 13 strains belonging to six DTUs such as Sylvio X10 cl1, Sylvio X10/4 and G strains for DTU-I, Y and Esmeraldo strains for DTU-II, M6241 cl6 and 3869 strains for DTU-III, CanIII cl1 and José Júlio strains for DTU-IV, MN cl2 and NR cl3 strains for DTU-V and CL Brener and CL14 for DTU-VI. The Tc-STAMS2 method was able to correctly discriminate intra-DTU strains with high accuracy (**Fig 4**). Indeed, each strain matched correctly the corresponding one included into the database. Moreover, we assembled a MS/MS spectral library using two biological replicates of the 13 *T*.*cruzi* strains associated to six DTUs (**Fig 5**). One independent replicate of each strain was used to match the spectral library using the Tc-STAMS2 method. Each strain matched correctly to the corresponding DTU with high accuracy (**Fig 5**).

**Table 4:** Spectral matching of different *T*.*cruzi* strains using the DiagnoProt software. Seven *T*.*cruzi* strains (Sylvio X10 cl1, Y, M6241 cl6, CanIII cl1, MN cl2, CL Brener and CL14) belonging to six DTUs were selected to test the Tc-STAMS2 method. The database was constructed with six strains (Sylvio X10 cl1, Y, M6241 cl6, CanIII cl1, MN cl2 and CL Brener) and it was used for comparison of the different strains including CL14.

DTUs	DTU-I	DTU-II	DTU-III	DTU-IV	DTU-V	DTU-VI
DTU-I	0.174	0.078	0.082	0.089	0.079	0.094
DTU-II	0.092	0.172	0.093	0.105	0.088	0.104
DTU-III	0.081	0.075	0.162	0.079	0.088	0.092
DTU-IV	0.095	0.084	0.082	0.155	0.079	0.089
DTU-V	0.070	0.065	0.077	0.063	0.155	0.095
DTU-VI	0.088	0.084	0.089	0.077	0.100	0.162
CL14	0.215	0.237	0.246	0.214	0.262	0.360

### Clustering analysis using peptide and protein identification

Database search was also performed to evaluate the similarity among the DTUs using peptide and protein identification results. Three database search platforms were used (MaxQuant, TPP and Proteome Discoverer). From four replicates of each DTU more than 7000 peptides and 4000 proteins were identified (**S5 Fig**, **S1 Table, S2 Table and S3 Table**). DTU-I and DTU-VI had the highest number of identifications due to the protein database used for the search. Indeed, Sylvio (DTU-I) and CL Brener (DTU-VI) are the two *T*.*cruzi* strains whose genome has been sequenced and their proteome annotated and deposited in the Uniprot database. Interestingly, only 30% of the MS/MS spectra were assigned, leaving behind a wealth of information for *T*.*cruzi* strain discrimination (**S4 Table**).

Analysis of variance (ANOVA p<0.05 followed by Benjamin-Hochberg FDR correction) was applied for the log2-transformed protein or peptide intensities previously identified using MaxQuant. A total of 1096 proteins and 6130 peptides showed significant difference in abundance among the six DTUs (**S5 Table**). The differentially expressed peptides and proteins were subjected to clustering analysis and visualized as heat-maps, **S6A and S6B Fig**. CL14 clustered together with CLBrener (DTU-VI), indicating high similarity in the protein and peptide expression profile.

**Fig 6:**
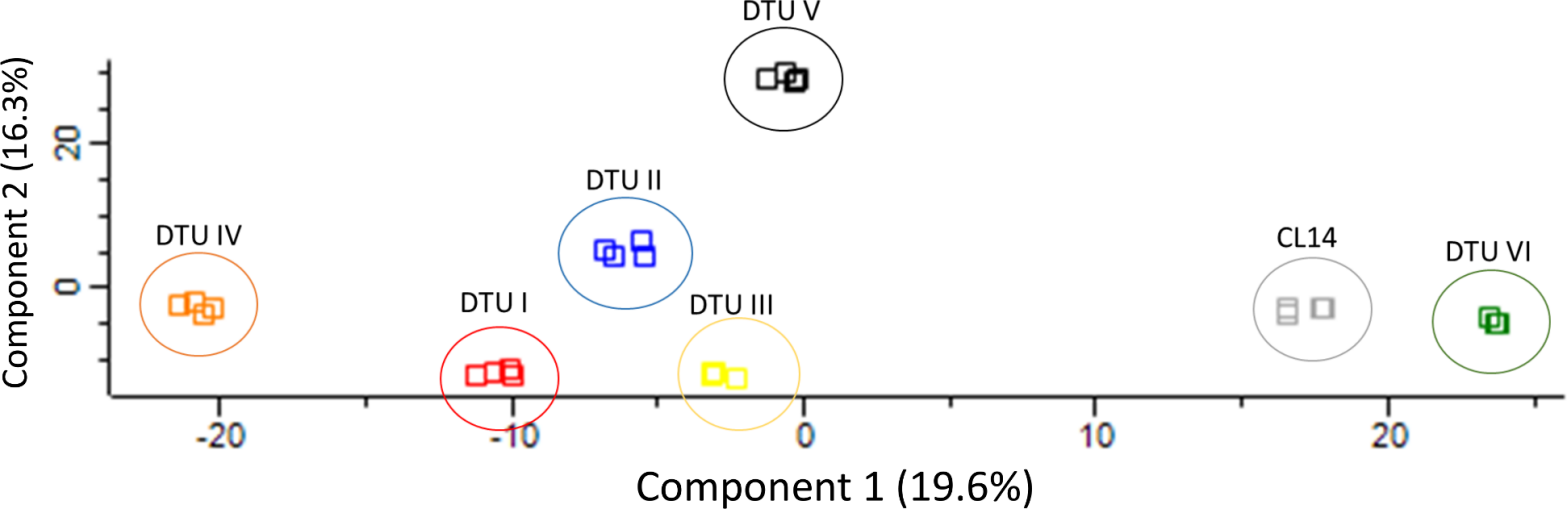
Principal component analysis of seven *T*.*cruzi* strains belonging to six DTUs based on differentially regulated proteins. LC-MS/MS data acquired for Sylvio X10 cl1 (DTU-I), Y (DTU-II), M6241 cl6 (DTU-III), CanIII cl1 (DTU-IV), MN cl2 (DTU-V), CL14 and CL Brener (DTU-VI) strains were searched against Uniprot *T*. *cruzi* Protein Database (release July 11, 2017; 51,738 entries) using MaxQuant computational platform as described in step C of Fig 1. Quantified proteins were analysed by ANOVA with Benjamin-Hochberg correction. 1096 differentially expressed proteins (qvalue<0.05) were used to perform the principal component analysis (PCA). DTU I—Red, DTU II—Blue, DTU III—Yellow, DTU IV—Orange, DTU V–Black, CLBrener–Green and CL14—Grey. All DTUs were separated and two strains belonging to DTU-VI (CL Brener and CL14) clustered together.

In addition, principal component analysis (PCA), which was applied in the differential expressed proteins, were able to discriminate the six DTUs showing a DTU-specific quantitative proteome repertoire **(Fig 6)**. Interestingly, CL14 and CLBrener, strains belonging to the DTU-VI, were also found close to each other, confirming the Euclidean clustering result obtained previously.

Additionally, to check the proteomic data reproducibility, a correlation analysis (R squared) between biological replicates from each DTU or between the different DTUs was performed using the log2-normalized intensities. As shown in **S7 Fig**, high correlation values were observed among replicates (R squared>0.9). Interestingly, when we compared different DTUs (**S8 Fig**), the R squared dropped to 0.6–0.7, but high correlation was observed between CL14 and DTU-VI (R squared = 0.83), confirming once more the similarity between these two DTUs (**S8 Fig**).

## Discussion

Many studies have employed MS-based techniques to identify organisms, such as bacteria [34]. Önder et, al. pioneered the use of MS/MS spectral libraries to precisely identify which animal the tick *Ixodes scapularis* was fed even if the feeding occurred months earlier [16]. Other strategies combined genetic information in conjunction with MS-based peptide identification for the correct assignment of microorganisms [35] [36, 37]. More recently, Shao et al. demonstrated that it is possible to identify *E*. *coli* strains with only the MS/MS fragmentation spectra, with no need for peptide identification (Shao et al. 2015).

In the present study, we describe a genome-free, MS/MS spectral-matching methodology designed to identify different *T*. *cruzi* DTUs, named Tc-STAMS2. Firstly, a peptide MS/MS spectral library using three replicates from each of the six DTUs was built. The fourth replicate was used to test the ability of the method to differentiate each DTUs using the reference library. As shown in **Tables 2 and 4 and Figs 5 and 6**, this approach was able to differentiate strains belonging to similar and different DTUs. A unique assignment to the correct DTU was achieved. The method was tested using a dataset of peptide MS/MS spectra obtained from different growth conditions of *T*.*cruzi* and no bias was detected.

The next step was to test the MS/MS spectral library against phylogenetically related species such as *T*.*vivax* and distant organisms such as *E*.*coli*, *mus musculus* and *homo sapiens* (**Fig 2**). For such a test, another spectral library database was built with the same DTUs and MS/MS spectra from the CL14 strain and *T*. *vivax*. Interestingly, it is clear that even with thousands of fragmentation spectra, the scores obtained when comparing samples of human organism or *E*.*coli* with the library were close to zero, demonstrating the specificity of the method in identifying only samples of the species/strain, whose MS/MS spectrum is present in the library.

We also showed that the spectral matching method is robust even with inter and intra-laboratory source of variations using similar MS instruments, but from different laboratory and even performing changes in the sample preparation, chromatography and fragmentation method, it was still possible to correctly identify samples from DTU-III and DTU-I, as shown in **Fig 3**.

As expected, we also observed that the similarity score is dependent on the number of MS/MS spectra acquired. The longer the gradient time used, the greater the separation capacity of peptides prior to MS/MS analysis and the larger the number of acquired spectra, resulting in higher scores for the same sample when compared to the library. Using different fragmentation methods we also observed that CID provides scores higher than HCD when using 70 min gradient time. Although HCD provides high resolution MS/MS spectra CID provides faster MS/MS sequencing, thus generating more spectra that can be match with the spectral library.

In addition, fragmentation spectra were also subjected to database search analysis for peptide and protein identification using the MaxQuant software, TPP and Proteome Discoverer. In general, the number of identifications of proteins and peptides were reproducible and consistent among different database search software. The larger number of proteins and peptides were observed for DTU-I and VI. Although the number of annotated proteins in UniProt database is greater for DTUs I and VI, the number of proteins identified to DTUs V, III and VI, was not significantly smaller. Due to that, a MS-based proteomic approach can be used to quantitatively compare the protein expression of different DTUs, even with differences in genome annotation among them and use these information’s to identify DTU-specific pathways correlated with the strain phenotype.

Moreover, it was possible to cluster together DTU-VI and CL14 using the differential expressed proteins or peptides given by ANOVA test, **S6 Fig**. This result validates which is already known in the literature, where the CL14 belong to DTU-VI [5] and also validates the results obtained with the spectral matching, where the similarity score between CL14 and DTU-VI was higher compared to the other DTUs. Moreover, PCA analysis was also able to discriminate the six DTUs and determine the similarity between the DTUVI and CL14, as seen by the close proximity between these two groups.

Clustering analysis using protein identification in different DTUs was also performed by *Telleria* et, al., [38] however only 261 (experiment 1) and 172 (experiment 2) 2DE protein spots were considered for this analysis. In our study, the clustering analysis was performed using 1096 proteins differentially expressed among DTUs, increasing the number of features to build a robust *T*. *cruzi* clustering (**S6 Fig**).

The Tc-STAMS2 strategy presented here is robust, accurate, easy to perform and completely automated. Dworzanski, J. P. [37] and Shao, W. et al. [15] used a similar methodology for identification of bacteria, however this is the first time that spectral matching is applied to discriminate different *T*. *cruzi* DTUs.

The transmission of Chagas disease through blood transfusion and organ transplant, besides the triatomine vector, poses several public health challenges. Due to that, novel methods identify and characterize DTUs can offer opportunities to understand the DTUs diversity, link with their phenotype and provide another tool for molecular epidemiology. Recently, MALDI-based strategy was developed for the direct identification of trypanosomatids based on the MS profile, named DIT-MALDI TOF [39]. However, this method does not have the resolution to discriminate DTUs using the MS profile. In this study we present a *T*. *cruzi* strain typing based on MS/MS spectral matching. The Tc-STAMS2 method is robust, sensitive and powerful and it is based on the identification of peptides from their MS/MS spectra. The method could be used to complement other already established methods. The proposed method can help in the research of epidemiology to identify *T*. *cruzi* strains only with the use of fragmentation spectra without the need for genomic data. In this study, we used the epimastigote form of *T*.*cruzi* to implement the Tc-STAMS2 method. However, quantitative proteomic analysis of *T*.*cruzi* epimastigote and trypomastigote life stages have shown distinct protein expression profile [40, 41]. Due to that, more studies are needed to confirm the specificity of the Tc-STAMS2 method using other *T*.*cruzi* stages. Since MS/MS spectra are available to the research community further data mining is possible in order to improve our understanding on the biology of the different *T*. *cruzi* strains. The methodology shown in this study will provide a complementary tool to the current nucleic-based testing and have the possibility to be extended to other parasitic diseases.

## Supporting information

S1 Fig LC-MS chromatographic profile of tryptic peptides belonging to the four replicates from Sylvio X10 cl1 (DTU-I). The Pearson correlation score is reported on the right side of the chromatogram and was calculated based on the quantified proteins in each replicate compared to the first replicate.
(TIF)

S2 Fig LC-MS chromatographic profile of peptides belonging to the six *T*.*cruzi* strains. (TIF)

S3 Fig LC-MS chromatographic profile of peptides belonging to the CLBrener and CL14 *T*.*cruzi* strains. Three replicates for each strain are reported.
(TIF)

S4 Fig LC-MS chromatographic profile of peptides belonging to the Sylvio X10 cl1 (DTU-I) acquired in the PR group, Odense, Denmark and in the CEFAP mass spectrometry facility at USP, Sao Paulo, Brazil (blind test). (TIF)

S5 Fig A. Peptide identifications using MaxQuant, TPP and Proteome Discoverer software for the Sylvio X10 cl1 (DTU-I), Y (DTU-II), M6241 cl6 (DTU-III), CanIII cl1 (DTU-IV), MN cl2 (DTU-V), CL Brener (DTU-VI). B. Protein identifications using MaxQuant, TPP and Proteome Discoverer software for the Sylvio X10 cl1 (DTU-I), Y (DTU-II), M6241 cl6 (DTU-III), CanIII cl1 (DTU-IV), MN cl2 (DTU-V), CL Brener (DTU-VI).
(TIF)

S6 Fig A) proteins and B) peptides differentially regulated between the different DTUs with q<0.05 were hierarchically clustered based on Euclidean distances using Perseus software.
(PPTX)

S7 Fig Multi scatter plot obtained comparing the quantified proteins of the six DTUs with four technical replicates each using all quantified proteins. A–Sylvio X10 cl1 (DTU-I), B—Y (DTU-II), C—M6241 cl6 (DTU-III), D—CanIII cl1 (DTU-IV), E—MN cl2 (DTU-V), F—CL Brener (DTU-VI) *T*.*cruzi* strains.
(TIF)

S8 Fig Multi scatter plot obtained comparing the quantified peptides of the Sylvio X10 cl1 (DTU-I), Y (DTU-II), M6241 cl6 (DTU-III), CanIII cl1 (DTU-IV), MN cl2 (DTU-V), CL Brener and CL14 (DTU-VI) *T*.*cruzi* strains. (TIF)

S1 Table Number of protein IDs using MaxQuant computational platform. Column BV with the header “Type” indicates the DTUs where each protein was identified.
(XLSX)

S2 Table Number of protein IDs using Trans-Proteomic Pipeline computational platform. Column K with the header “Type” indicates the DTUs where each protein was identified.
(XLSX)

S3 Table Number of protein IDs using Proteome Discoverer computational platform. Column AB with the header “Type” indicates the DTUs where each protein was identified.
(XLSX)

S4 Table Number of PSMs and unannotated MS/MS spectra. Protein accessions are organized based on the DTU where they were identified.
(XLSX)

S5 Table Regulated proteins and peptides among the six DTUs. Protein accessions are organized based on the DTU where they were identified.
(XLSX)
