# Supplementary figures and images for: Development of a Trypanosoma cruzi strain typing assay using MS2 peptide spectral libraries (Tc-STAMS2)
Source: PLoS Negl Trop Dis. 2018 Apr 2;12(4):e0006351. doi: 10.1371/journal.pntd.0006351 (PMC5897034; doi:10.1371/journal.pntd.0006351)

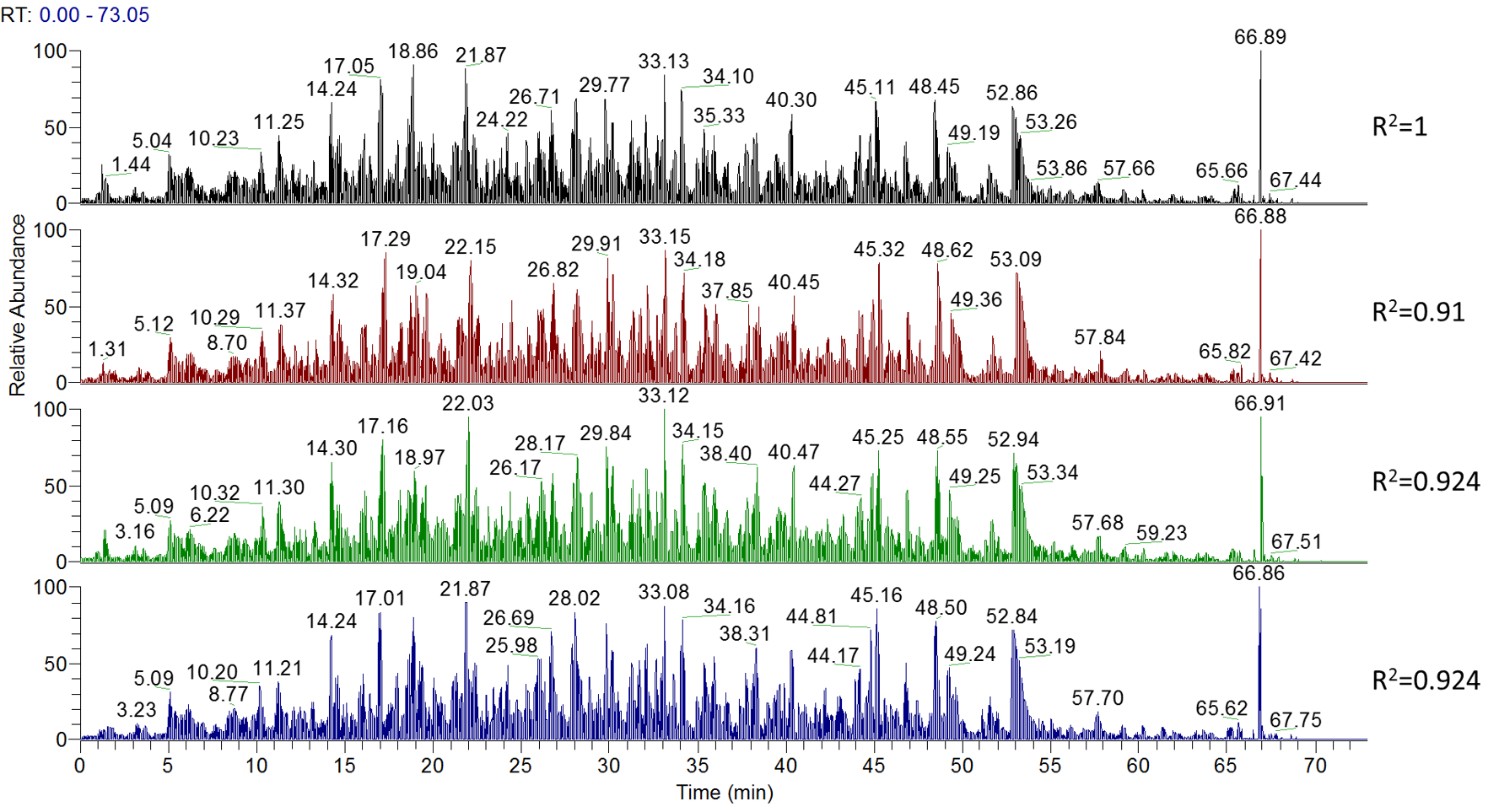

Supplement: S1 Fig — The Pearson correlation score is reported on the right side of the chromatogram and was calculated based on the quantified proteins in each replicate compared to the first replicate. (TIF) [file pntd.0006351.s001.tif]

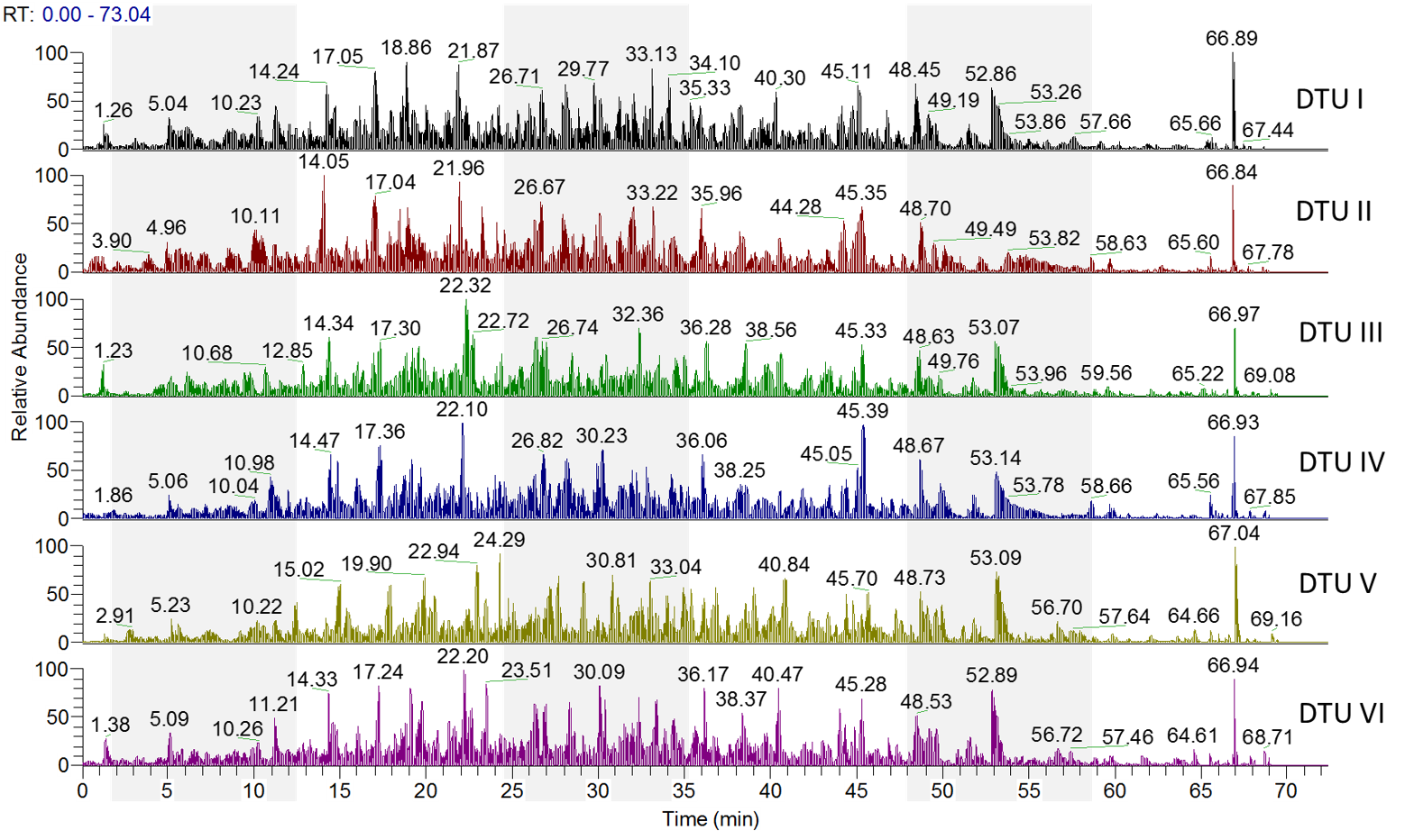

Supplement: S2 Fig — (TIF) [file pntd.0006351.s002.tif]

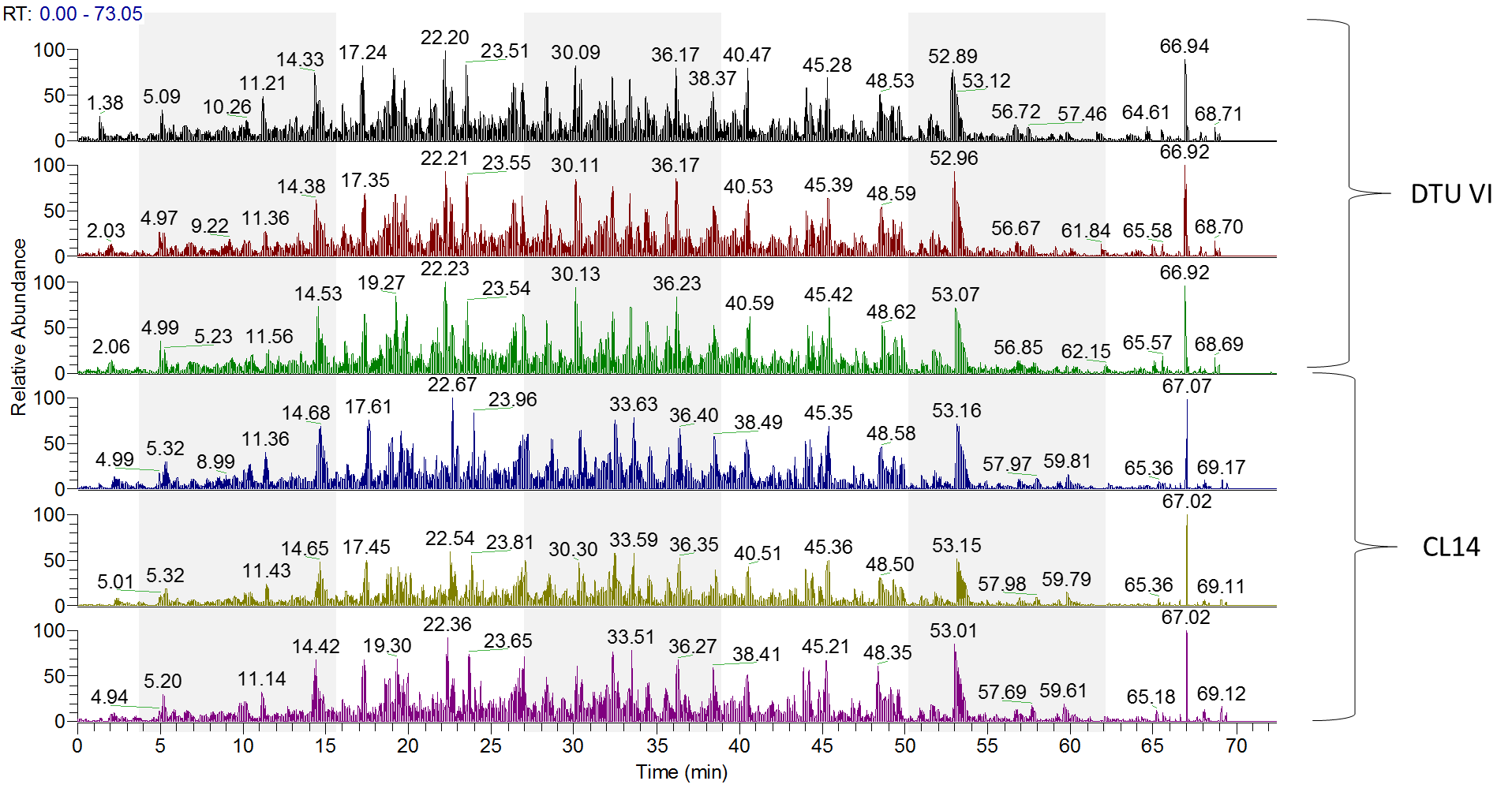

Supplement: S3 Fig — Three replicates for each strain are reported. (TIF) [file pntd.0006351.s003.tif]

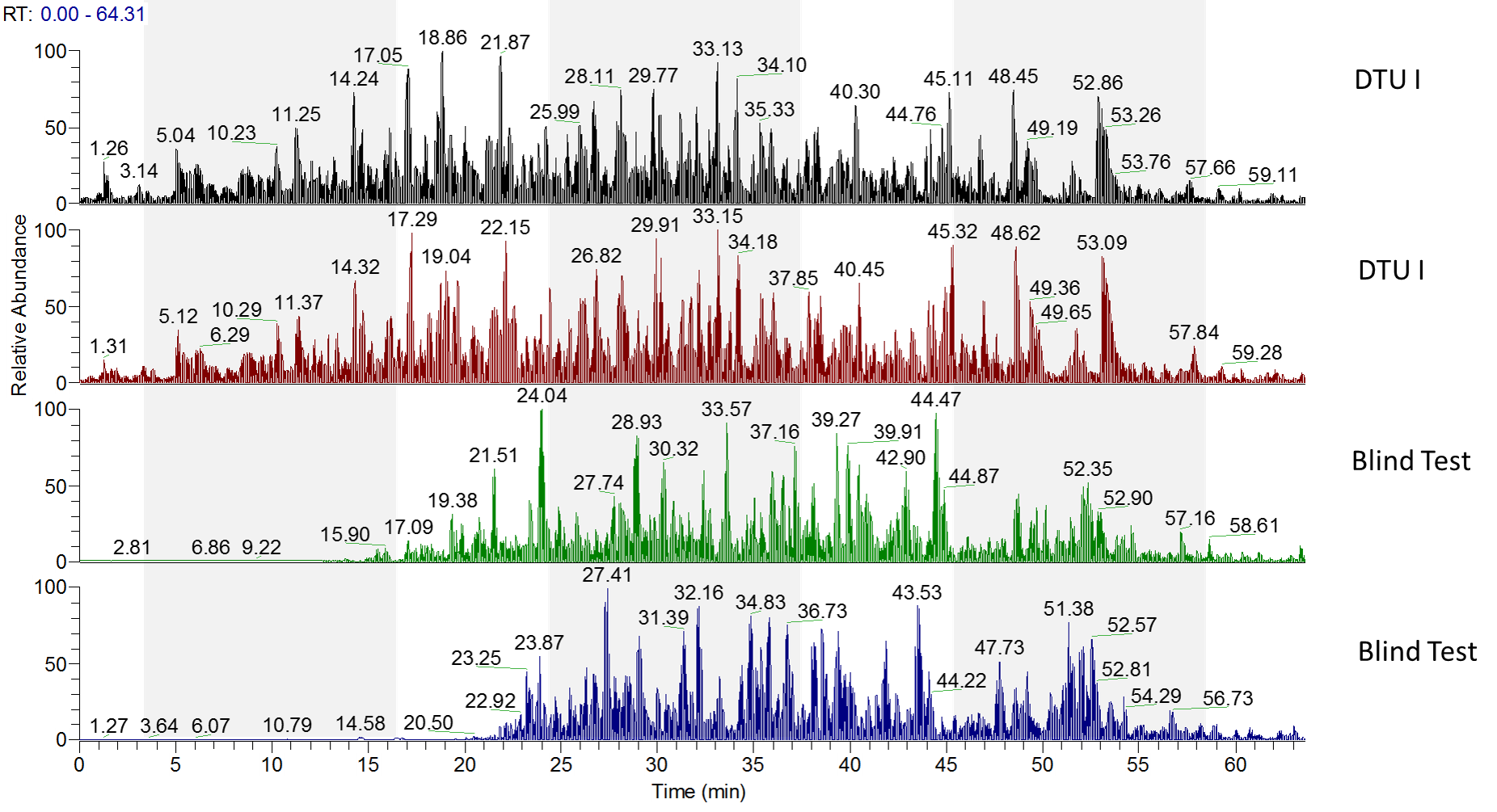

Supplement: S4 Fig — (TIF) [file pntd.0006351.s004.tif]

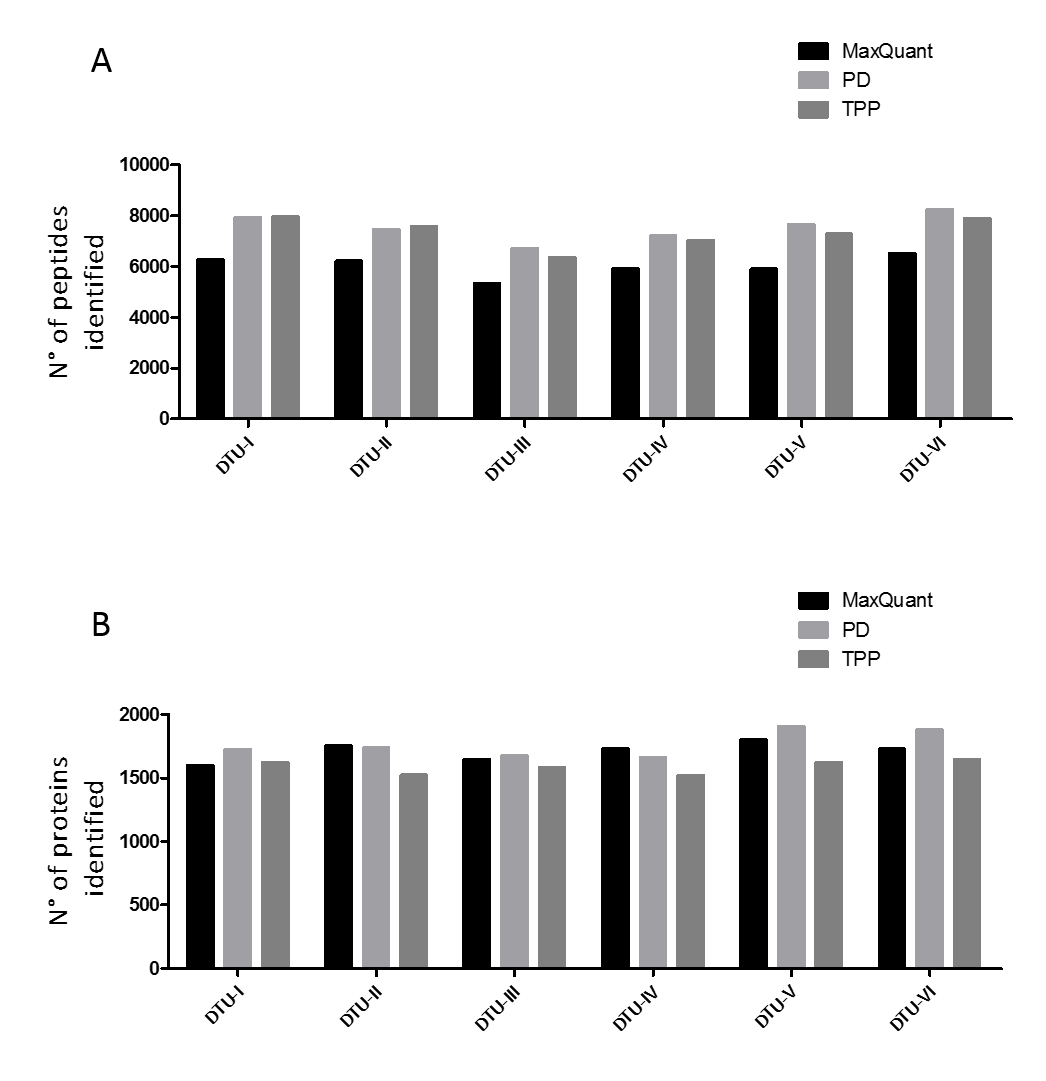

Supplement: S5 Fig — A. Peptide identifications using MaxQuant, TPP and Proteome Discoverer software for the Sylvio X10 cl1 (DTU-I), Y (DTU-II), M6241 cl6 (DTU-III), CanIII cl1 (DTU-IV), MN cl2 (DTU-V), CL Brener (DTU-VI). B. Protein identifications using MaxQuant, TPP and Proteome Discoverer software for the Sylvio X10 cl1 (DTU-I), Y (DTU-II), M6241 cl6 (DTU-III), CanIII cl1 (DTU-IV), MN cl2 (DTU-V), CL Brener (DTU-VI). (TIF) [file pntd.0006351.s005.tif]

## Slide 1
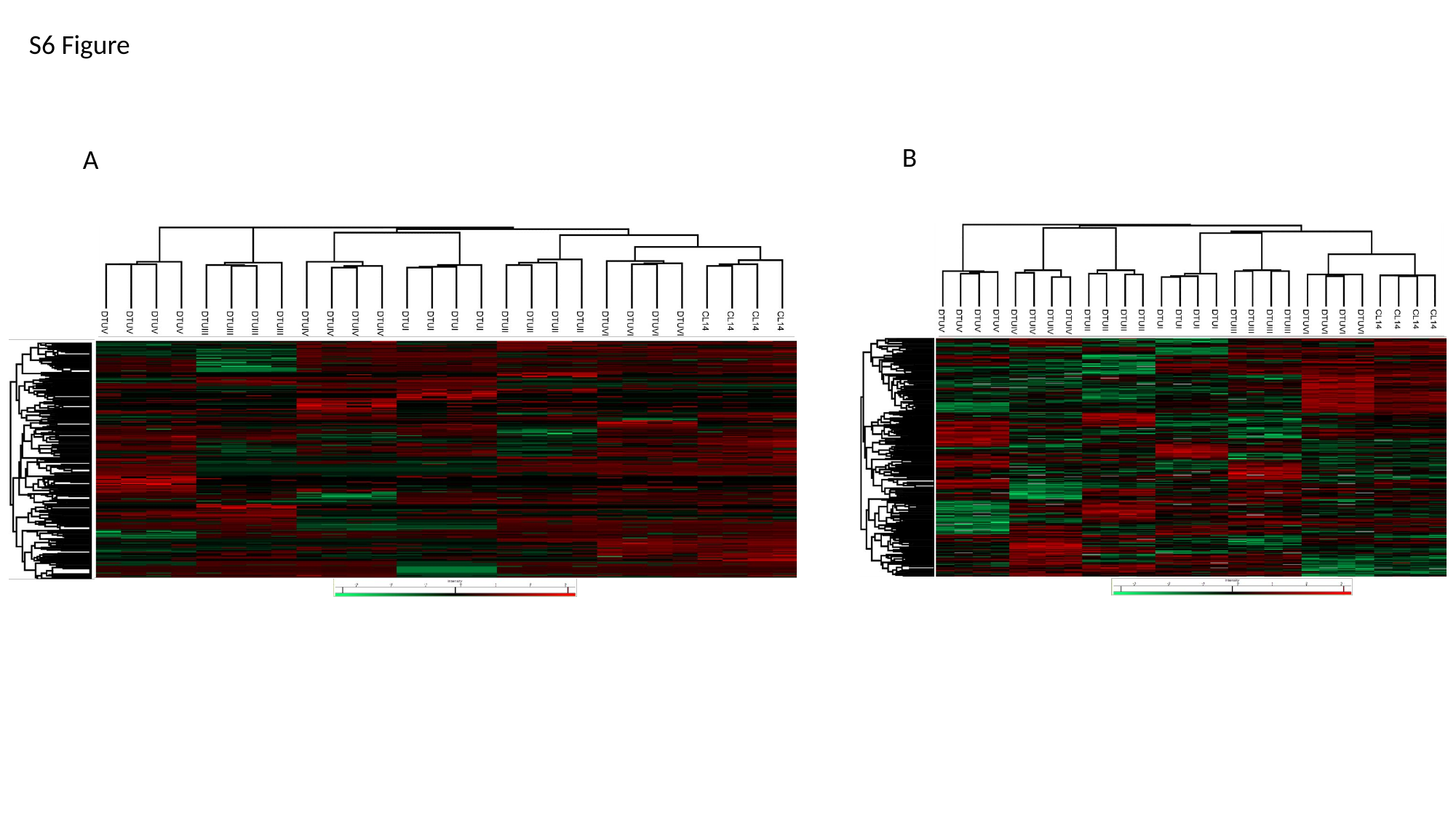

S6 Figure
B
A

Supplement: S6 Fig — A) proteins and B) peptides differentially regulated between the different DTUs with q<0.05 were hierarchically clustered based on Euclidean distances using Perseus software. (PPTX) [file pntd.0006351.s006.pptx]

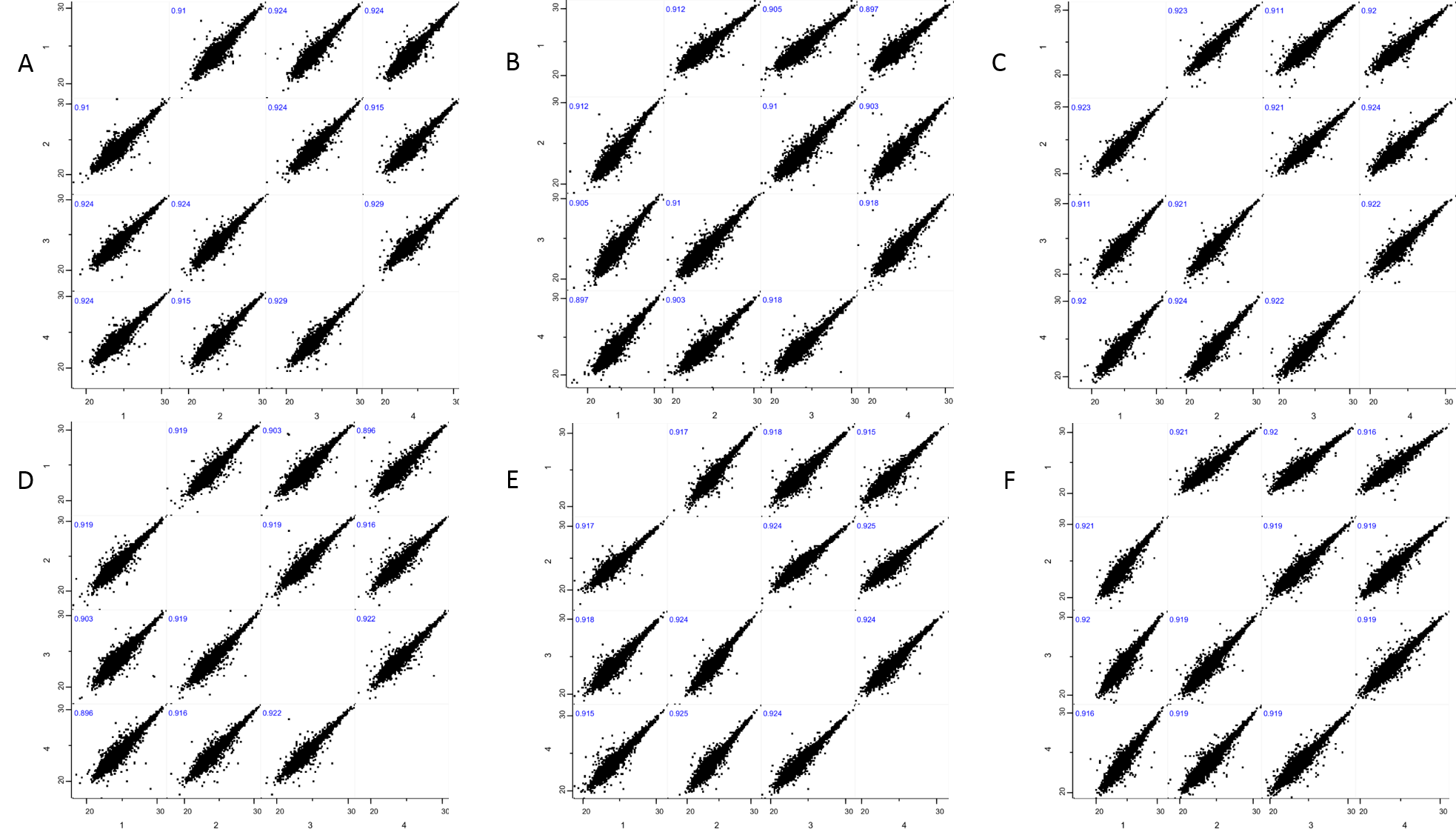

Supplement: S7 Fig — A–Sylvio X10 cl1 (DTU-I), B—Y (DTU-II), C—M6241 cl6 (DTU-III), D—CanIII cl1 (DTU-IV), E—MN cl2 (DTU-V), F—CL Brener (DTU-VI) T.cruzi strains. (TIF) [file pntd.0006351.s007.tif]

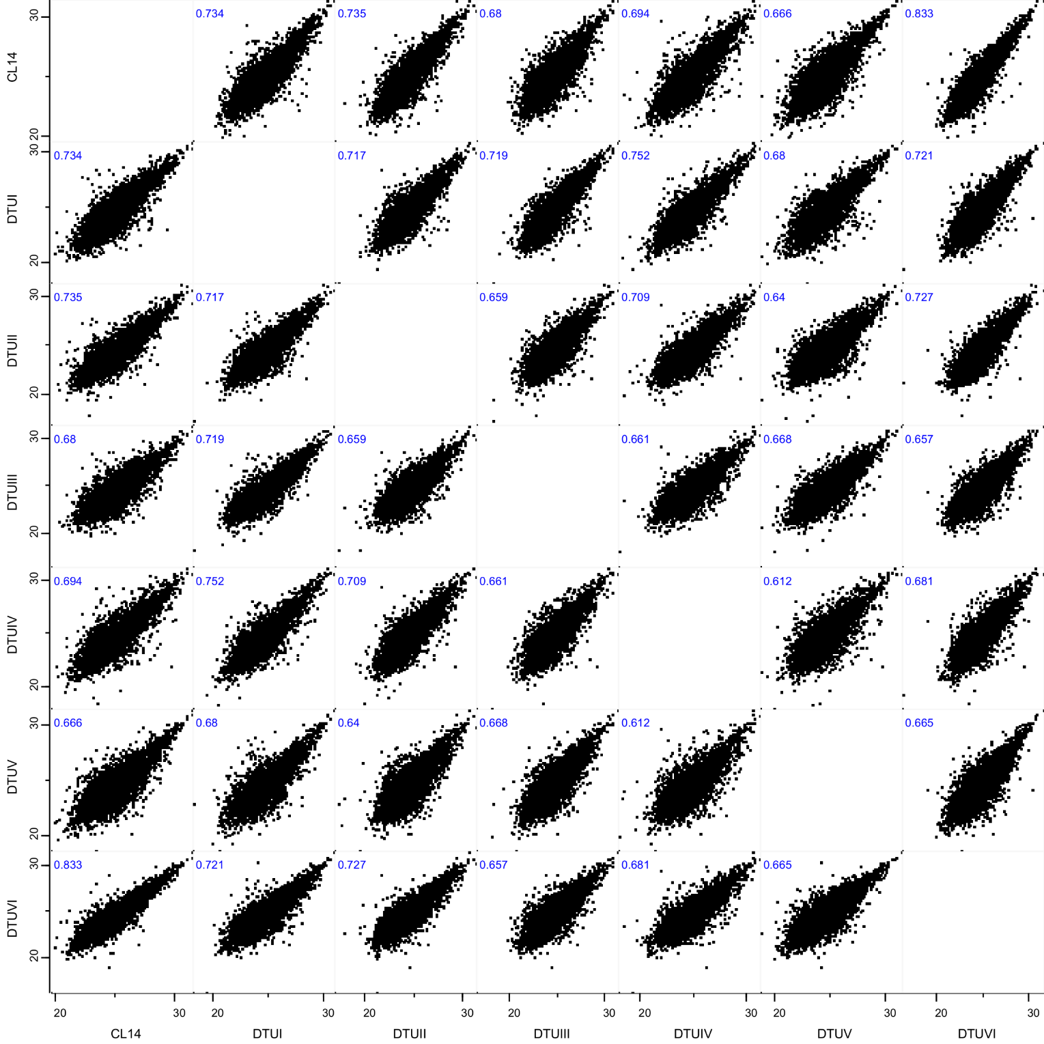

Supplement: S8 Fig — (TIF) [file pntd.0006351.s008.tif]
